# Prognostic significance of neutrophil to lymphocyte ratio in ovarian cancer: evidence from 4,910 patients

**DOI:** 10.18632/oncotarget.20196

**Published:** 2017-08-10

**Authors:** Quan Zhou, Li Hong, Man-Zhen Zuo, Ze He

**Affiliations:** ^1^ Department of Gynecology and Obstetrics, The People’s Hospital of Three Gorges University/The First People’s Hospital of Yichang, Yichang 443000, P. R. China; ^2^ Department of Gynecology and Obstetrics, Renmin Hospital of Wuhan University, Wuhan, Hubei 430060, P. R. China

**Keywords:** neutrophil to lymphocyte ratio, ovarian cancer, prognostic value

## Abstract

Increasing evidence indicates that elevated neutrophil to lymphocyte ratio (NLR) are related with poor prognosis in various types of tumors. However, the prognostic role of NLR in patients with ovarian cancer (OC) remains controversial. Thus, the current meta-analysis aimed to investigate the prognostic role of NLR in patients with OC. A total of 16 studies with 4,910 patients were included. By pooling hazard ratios (HRs) with 95% confidence intervals (CIs) and odds ratios (ORs) with 95% CIs from each study. The results demonstrated that elevated pretreatment NLR was significantly related to poor OS (HR: 1.50, 95% CI: 1.27-1.77) and PFS (HR: 1.53, 95% CI: 1.28-1.84) in patients with OC. Subgroup analyses was divided by ethnicity, sample size, histologic types, cut-off value of NLR, analysis method and NOS score, but the results did not showed any significant change the main results. This meta-analysis revealed that elevated pretreatment NLR might be a predicative factor of poor prognosis in OC patients.

## INTRODUCTION

Ovarian cancer (OC) is the second most common female genital tract cancer and the most lethal form of all gynecological malignancies [1]. Based on GLOBOCAN estimates, more than 238,700 new cases and 151,900 women died from ovarian cancer every year in worldwide [2]. Due to the lack of specific symptoms and efficiently prognostic biomarkers, over 75% of patients are at advanced stage of the disease (Stage III or IV) when being diagnosed [3, 4]. About 30%-40% of OC patients can survive for 5 years while 10-year survival rate is only 5%-21% [4, 5]. Therefore, efficient and reliable biomarkers for individualized prediction of treatment outcomes and prognosis in early stage of OC patients are urgently required.

Accumulating evidence has shown that inflammation plays an essential role in tumor development at different stages including initiation, promotion, malignant conversion, invasion, and metastasis. The systemic inflammation is associated with poor prognosis in most of cancers [6–8]. The prognostic value of systemic inflammatory response markers has received paramount attention, and a variety of blood-based parameters that reflect the status of systemic inflammation have been extensively explored as prognostic biomarkers in various cancers including OC [9–11]. These inflammatory markers include C-reactive protein (CRP)[12], platelet count [13], neutrophil to lymphocyte ratio (NLR)[14], lymphocyte to monocyte ratio(LMR)[15], platelet to lymphocyte ratio (PLR)[16] and modified Glasgow prognostic score (mGPS)[9].

Among these inflammatory markers, the neutrophil-to-lymphocyte ratio (NLR) has gained notable interest [14, 17]. Increasing evidence indicated that NLR had prognostic significance in patients with different cancers including cervical cancer [18, 19], breast cancer [20, 21], thyroid cancer [22], lung cancer [23], colorectal cancer [24], prostate cancer [25], hepatocellular cancer [26], renal cancer [27], gastric cancer [28], pancreatic cancer [29], esophageal cancer [30, 31] and skin cancer [32]. Recently, several studies have evaluated the prognostic significance of NLR in OC patients; however, the majority of these studies had relatively limited sample sizes. Moreover, some authors presented inconsistent results due to the variances in study design, sample size and patient characteristics [33–48]. Meta-analysis is an effectively analytic approach to pool different studies to overcome above deficiencies and to provide more reliable results. We thus conducted a systematic review and meta-analysis to reveal the prognostic value of NLR in patients with OC.

## RESULTS

### Selection and characteristics of included studies

**Figure 1:**
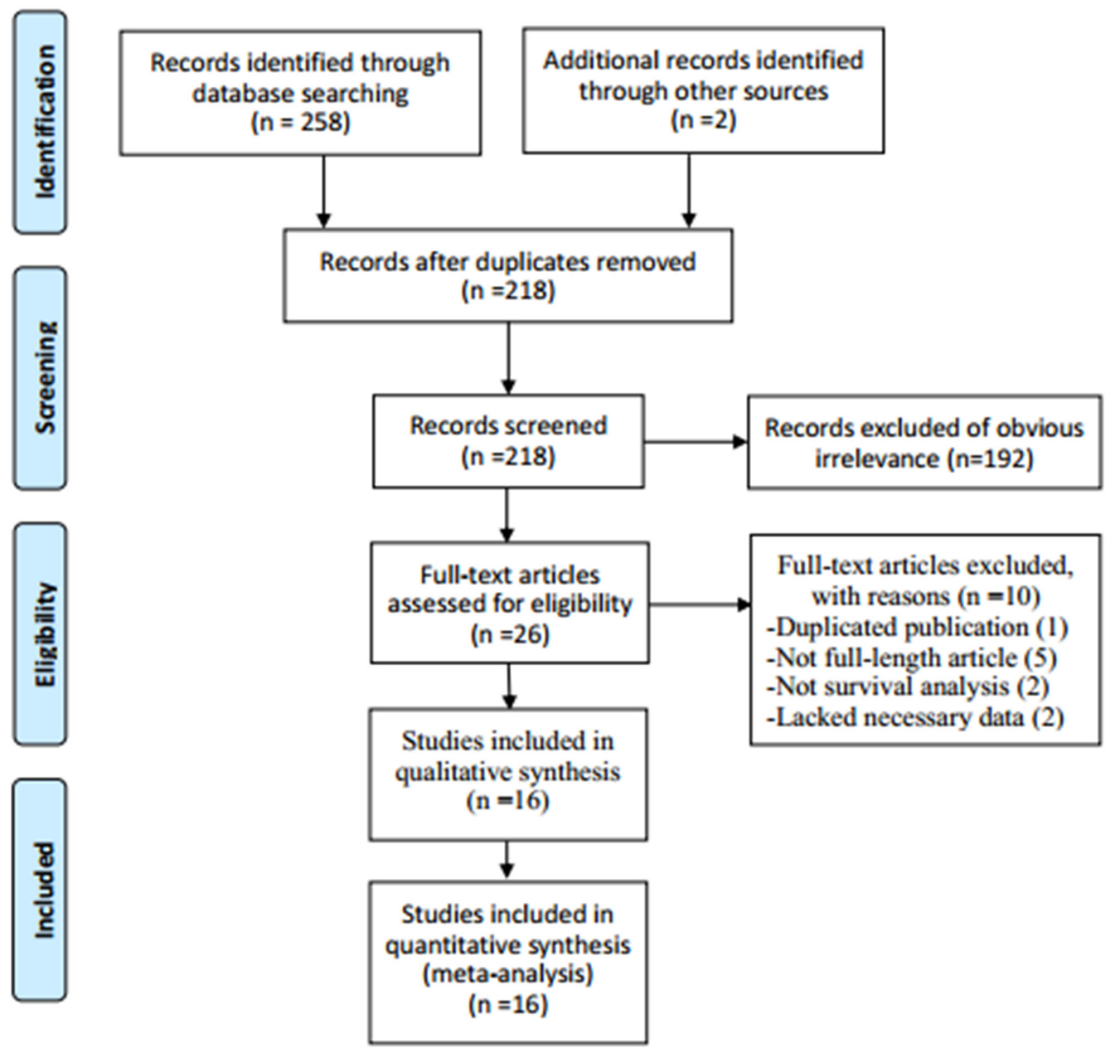
Flow chart showing the process for selecting eligible studies in the meta-analysis

The literature retrieval procedure was illustrated in Figure 1. The initial search collected a total of 260 articles (258 from database search, 2 from other sources), of which 42 duplicate publications were removed. The remaining 218 articles were subjected to next evaluation. After reviewing the titles and abstracts, 192 articles were excluded as they were duplicate reports, abstracts, reviews, case reports or studies irrelevant to the current analysis. Subsequently, the full text of remaining 26 articles was carefully reviewed to assess the eligibility of these articles and 10 articles were further excluded: 1 study had significant overlap, 5 articles were abstracts without detailed data, 2 papers were not prognostic article and 2 papers lacked necessary data for estimating the HR and 95% CI or the cut-off of NLR. Finally, 16 studies were used for the meta-analysis.

The main characteristics of the included studies were shown in Table 1. Total 4,910 patients from 16 studies published between 2009 and 2016 were included in the meta-analysis [33–48]. Among the 16 studies, 11 studies were conducted in Asian populations [35–37, 39, 41–45, 47, 48] while 5 studies were performed in Caucasian population [33, 34, 38, 40, 46], which included the following geographical regions: USA (n=3), Poland (n=1), South Korea (n=4), China (n=6), Turkey(n=1) and Thailand (n=1). The sample sizes in each study ranged 90 to 875. All included studies investigated the prognostic value of NLR for OS [33–48] and 12 studies investigated the prognostic role of NLR for PFS in the meta-analysis [34, 36–45, 48]. The median follow-up length of the studies was 52.7 months (range 29–93.7). The cut-off values applied by the included studies varied from 2.30 to 5.25, with a mean value of 3.37. 15 of the 16 studies used multivariate analysis. The scores of study quality estimated using the Newcastle Ottawa Scale (NOS) for quality assessment ranged from 5 to 8, with a mean value of 6.56.

**Table 1:** Main characteristics of all the studies included in the meta-analysis

Study cohort	Year	country	Ethnicity	Duration	No. patients	Age (years)	Histology	Stage	Grade	Treatment	Follow-up (momths)	Cut-off value	HR	Outcome	NOS score
Asher V [33]	2011	USA	Caucasian	1988-1998	235	NR	Mixed	I-IV	1-3	S+C	60	4	U/M	OS	8
Badora-Rybicka A [34]	2016	Poland	Caucasian	2007-2013	315	54(22-77)	Epithelial	I-IV	1-3	S+C	93.7	2.3	M	OS,PFS	6
Cho H [35]	2009	South Korea	Asian	2003-2006	192	52 (NA)	Epithelial	I-IV	1-3	S+C	20.9	2.6	U/M	OS	7
Eo WK [36]	2015	South Korea	Asian	2006-2013	234	56(14-84)	Epithelial	I-IV	1-3	S+C	60	4.28	U	OS,PFS	6
Feng Z [37]	2016	China	Asian	2005-2013	875	53 (30-90)	Serous	I-IV	1-3	S+C	29	2.6	M	OS,PFS	7
Gungorduk K [38]	2015	Turkey	Caucasian	1996-2011	91	57 (32-81)	PFTC	I-IV	1-3	S+C	34	2.7	U/M	OS,PFS	5
Kim HS [39]	2015	South Korea	Asian	1997-2012	109	53 (30-86)	Clear Cell	NR	NR	S+C	46	2.8	U/M	OS,PFS	7
Li Z [40]	2017	USA	Caucasian	2000-2010	654	63 (28-93)	Epithelial	I-IV	1-3	S+C	60	5.25	U/M	OS,PFS	8
Miao Y [41]	2016	China	Asian	2005-2010	344	55 (45-84)	Epithelial	I-IV	1-3	S+C	72	3.02	U/M	OS,PFS	6
Paik E [42]	2016	Korea	Asian	2002-2012	674	51 (15-84)	Epithelial	I-IV	1-3	S+C	52.5	3.91	U/M	OS, PFS	7
Thavaramara T [43]	2011	Thailand	Asian	2004-2009	129	50 (NA)	Epithelial	I-IV	1-3	S+C	NG	2.6	M	OS, PFS	6
Wang Y [44]	2015	China	Asian	2009-2010	126	NR	Serous	I-IV	1-3	S+C	41.3	3.77	U/M	OS,PFS	7
Wang YQ [45]	2016	China	Asian	2006-2013	143	52 (NA)	Mixed	I-IV	1-3	S+C	60	3.43	U/M	OS,PFS	5
Williams KA [46]	2014	USA	Caucasian	1992-2013	519	NR	Mixed	I-IV	1-3	S+C	68	3.6	U/M	OS	6
Zhang WQ [47]	2014	China	Asian	2007-2009	80	55 (27-83)	Epithelial	I-IV	1-3	S+C	45	3.8	U/M	OS	7
Zhang WW [48]	2015	China	Asian	2000-2012	190	51 (24-76)	Mixed	I-IV	1-3	S+C	43	3.4	U/M	OS,PFS	7

HR: hazard ratio; NOS: Newcastle Ottawa Scale; NR: not reported; S: Surgery; C: Chemotherapy; U: univariate; M: multivariate; OS: overall survival; PFS: Progression free survive; PFTC: primary fallopian tube carcinoma.

### NLR and OS in OC

**Figure 2:**
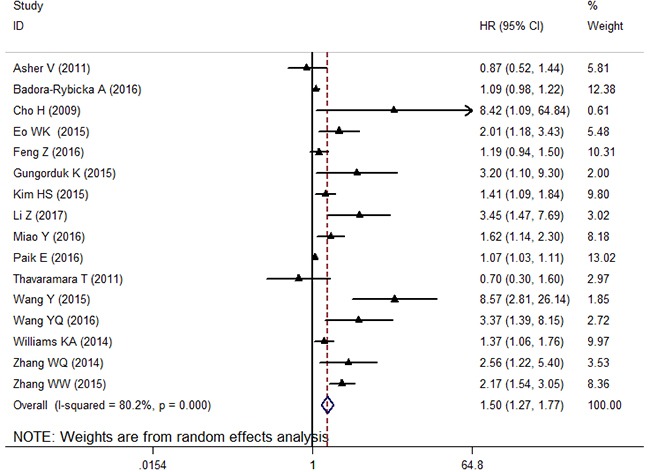
The forest plot between elevated NLR and OS in patients with OC

Total 16 studies with 4,910 OC patients evaluated the prognostic significance of NLR for OS. Meta-analysis of these 16 retrospective cohorts showed that the patients with elevated NLR were associated with unfavorable OS (HR: 1.50, 95% CI: 1.27-1.77, *p* < 0.001) (Figure 2). Due to the extreme heterogeneity between studies (*I^2^*: 80.2%, *P_h_* < 0.001), we conducted subgroup analyses according to the potential confounders, such as ethnicity, sample size, histologic types, cut-off value of NLR and NOS score. Subgroup analyses by ethnicity indicated that elevated NLR predicted poor prognosis for patients in Asian populations (HR: 1.69 95%CI: 1.31-2.21, random effects), but did not have prognostic efficiency for patients in Caucasian populations (HR: 1.36, 95%CI: 0.99-1.87, random effects). Stratification by sample size, the prognostic role of elevated NLR in predicting shorter OS was obvious not only in studies with small sample size (< 200) (HR: 2.29, 95% CI: 1.49-3.52, random effects), but also in studies with large sample (≥200) (HR: 1.23, 95% CI: 1.07-1.41, random effects). Furthermore, when analyzed based on histologic types, the data showed that elevated NLR indicated poor OS in patients with epithelial histologic types (HR: 1.28, 95% CI: 1.09-1.50, random effects) and mixed histologic types (HR: 1.93, 95% CI: 1.35-2.77, random effects). In addition, when the included cohorts were stratified by cut-off value of NLR, the data showed that the elevated NLR predicted prognosis for OC regardless of the cut-off value of NLR<3.4 (HR: 1.30, 95% CI: 1.05-1.61, random effects) and NLR≥3.4(HR: 1.89, 95% CI: 1.35-2.65, random effects). Finally, subgroup analyses by the NOS score showed that a high NLR indicated poorer OS in OC patients for studies with both NOS score ≥7 (HR: 1.49, 95% CI: 1.24-1.68, random effects) and NOS score< 7 (HR: 1.66, 95% CI: 1.24-2.22, random effects).

### NLR and PFS in OC

**Figure 3:**
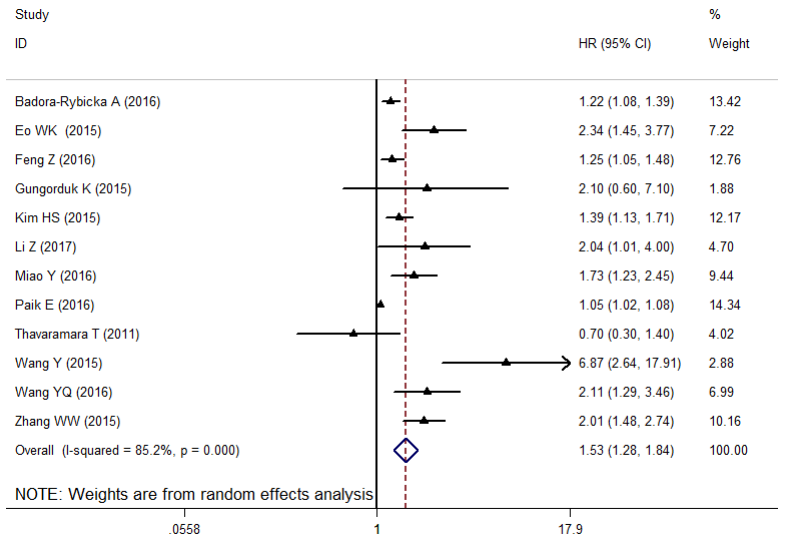
The forest plot between elevated NLR and PFS in patients with OC

Twelve cohorts with 3,884 subjects reported the data of relationship between NLR and PFS in patients with OC. The pooled result showed that increased NLR was significantly correlated with worse PFS (HR: 1.53, 95% CI: 1.28-1.84, *p* < 0.001), with extreme heterogeneity (*I^2^*: 85.2%, *P_h_* < 0.001) (Figure 3). We also explored the heterogeneity by conducting subgroup analyses. In the subgroup analysis based on ethnicity, an elevated NLR appeared to be a poor predictor for PFS no matter the patients were Caucasian (HR: 1.25, 95% CI: 1.10-1.42, Fixed) and Asian (HR: 1.59, 95% CI: 1.25-1.84, Random). Stratification by sample size, the obvious relationship between elevated NLR and poor PFS was found in studies with both small sample size (< 200) (HR: 1.83, 95% CI: 1.24-2.69, random effects) and large sample (≥200) (HR: 1.35, 95% CI: 1.13-1.62, random effects). Similarly, this trend was also observed with the stratification of histology type, cut-off value of NLR, analysis method and NOS score (Table 2).

**Table 2:** Summary of meta-analysis results

Factors	No. of studies	No. of patients	Effects model	HR(95% CL)	*P*	Heterogeneity	
						*I^2^*(%)	*P*_h_
**Overall survival (OS)**							
Overall	16	4910	Random	1.50 (1.27-1.77)	< 0.001	80.2%	< 0.001
Ethnicity:							
Caucasian	5	1269	Random	1.36 (0.99-1.87)	0.058	72.0%	0.006
Asian	11	3641	Random	1.69 (1.31-2.21)	< 0.001	83.5%	< 0.001
Sample size:							
<200	8	1605	Random	2.29 (1.49-3.52)	< 0.001	69.4%	0.002
≥200	8	3305	Random	1.23 (1.07-1.41)	0.002	69.6%	0.002
Histologic types:							
Epithelial	8	2622	Random	1.28 (1.09-1.50)	0.003	71.8%	0.001
Mixed	8	2288	Random	1.93 (1.35-2.77)	< 0.001	77.9%	< 0.001
Cut-off value:							
<3.4	7	2600	Random	1.30 (1.05-1.61)	0.014	60.9%	0.018
≥3.4	9	2310	Random	1.89 (1.35-2.65)	< 0.001	86.2%	< 0.001
NOS score:							
<7	7	1775	Random	1.49 (1.24-1.68)	0.005	70.7%	0.002
≥7	9	3135	Random	1.66 (1.24-2.22)	0.001	84.5%	< 0.001
Progression free survival (PFS)							
Overall	12	3884	Random	1.53 (1.28-1.84)	< 0.001	85.2%	< 0.001
Ethnicity:							
Caucasian	3	515	Fixed	1.25 (1.10-1.42)	0.001	27.5%	0.252
Asian	9	3369	Random	1.59 (1.25-1.84)	< 0.001	88.0%	< 0.001
Sample size:							
<200	6	1333	Random	1.83 (1.24-2.69)	0.002	73.1%	< 0.001
≥200	6	2551	Random	1.35 (1.13-1.62)	0.001	83.0%	< 0.001
Histology type:							
Epithelial	6	2350	Random	1.32 (1.09-1.59)	0.005	83.5%	< 0.001
Non-epithelial	6	1534	Random	2.07 (1.39-3.01)	< 0.001	75.4%	0.001
Cut-off value:							
<3.4	6	2408	Fixed	1.28 (1.17-1.40)	< 0.001	29.9%	0.211
≥3.4	6	1476	Random	2.09 (1.30-3.37)	< 0.001	90.5%	< 0.001
Analysis method:							
Univariate	2	377	Fixed	1.09 (1.06-1.12)	< 0.001	0.0%	0.768
Multivariate	10	3507	Random	1.43 (1.19-1.71)	< 0.001	84.4%	< 0.001
NOS score:							
<7	6	1256	Random	1.57 (1.15-2.15)	0.005	68.4%	0.007
≥7	6	2628	Random	1.54 (1.18-2.01)	0.001	88.8%	< 0.001

*P* denotes P value for statistical significance based on Z test; *P*h denotes *P* value for heterogeneity based on Q test. HR hazard ratio; CI confidence interval.

### Meta-regression analysis

Heterogeneity among studies was observed in the overall comparisons as well as in the subgroup analyses. We conducted meta-regression analysis by using variables as ethnicity (Asian vs. Caucasian), sample size (<200 vs.≥200), histologic types (Epithelial vs. Mixed), cut-off value of NLR (<3.4 vs.≥3.4) and NOS score (<7 vs.≥7), to investigate the potential source of heterogeneity among studies for OS and PFS. In multivariate analysis, the results showed that ethnicity (*P* = 0.744), sample size (*P* = 0.074), histologic types (*P* = 0.278), cut-off value of NLR (*P* = 0.526) and NOS score (*P* = 0.465) did not contribute to the source of heterogeneity for OS. Moreover, the data demonstrated that ethnicity (*P* = 0.884), sample size (*P* = 0.585), histologic types (*P* = 0.346), cut-off value of NLR (*P* = 0.585), analysis method (*P* = 0.839) and NOS score (*P* = 0.785) were not associated for the source of heterogeneity for PFS.

### Sensitivity analysis

**Figure 4:**
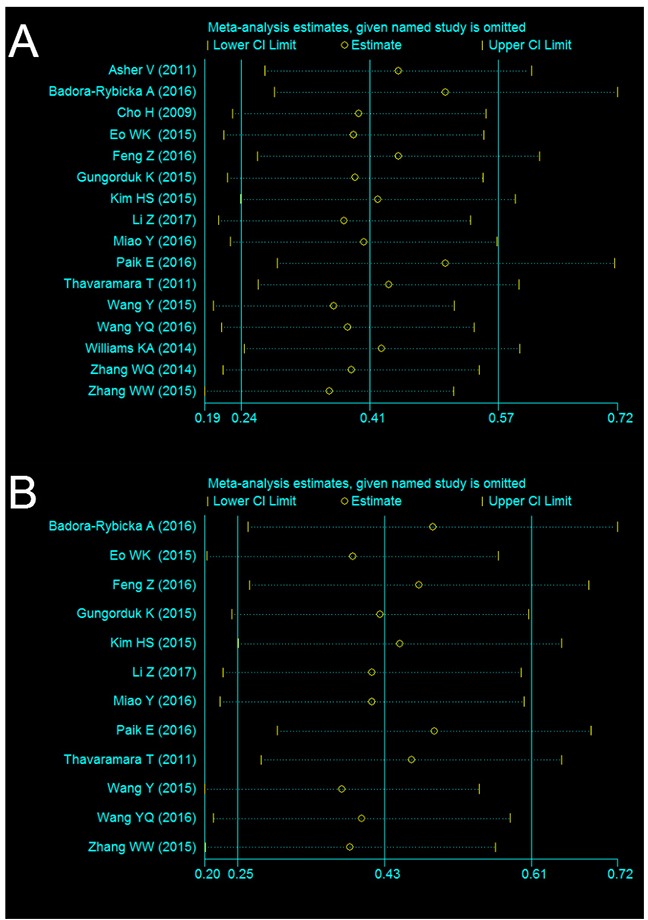
Sensitivity analysis of NLR on OS **(A)** and PFS **(B)** in OC patients.

By omitting the included studies sequentially, a sensitivity analysis was performed to assess the influence of the each included study on the pooled HR on OS and PFS was performed. The pooled HRs for OS and PFS were not substantially change. The results showed that the pooled HRs were stable and robust. The results of the sensitivity analysis were shown in Figure 4.

### Publication bias

**Figure 5:**
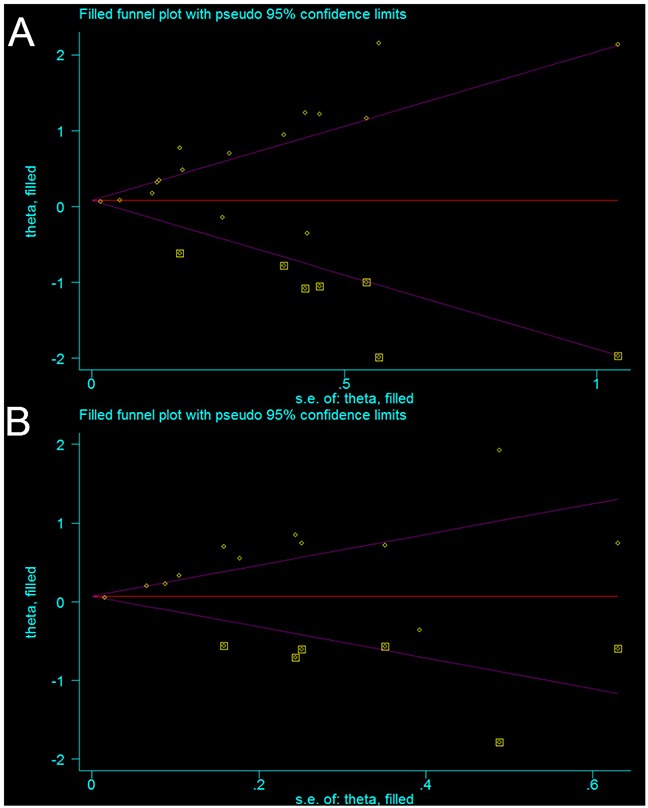
Funnel plot adjusted using the trim and fill method for OS **(A)** and PFS **(B)** in patients with OC. Circles: included studies. Diamonds: presumed missing studies.

Begg’s funnel plot and Egger’s test were performed to evaluate the publication bias of the included studies. The Begg’s funnel shape of the funnel plots showed that the dots were not symmetrically distributed(Figure 5), and the results of Egger’s test demonstrated that there was significant publication bias in OS and PFS (*P>|t|* = 0.000 for OS and *P>|t|* = 0.001 for PFS, respectively). The results revealed publication bias in this meta-analysis. Therefore, we further performed a “trim and fill” analysis to identify the source of the publication bias. It was estimated that there were seven unpublished studies evaluating the role of the NLR in OS and six unpublished studies evaluating the role of NLR in PFS (Figure 5). The recalculated results that combined estimated unpublished studies did not change significantly for OS (HR: 1.08, 95%CI: 1.05–1.12, *p* < 0.001) and PFS (HR = 1.07, 95%CI: 1.04-1.11, *p* < 0.001), indicated a positive outcome even though publication bias still exists.

## DISCUSSION

Nowadays, many prognostic factors have been investigated in an attempt to improve treatment outcomes in OC patients, such as age, FIGO stage, residual tumor, lymph node metastasis, vascular invasion and resection margin involvement. However, these factors have some limitations including difficulty in obtaining tumor samples, time consuming, high cost to identify, and insufficient specificity or sensitivity for specific OC, which limit their extensive application in clinical settings [4, 5, 34, 35]. Therefore, new clinical predictive and prognostic markers are required. The strong linkage between inflammation and cancer progression has been well established in the past decade [6, 8, 10]. Interestingly, recent studies demonstrated that inflammation-related neutrophils and immunocytes mediate the communication between tumor cells and tumor microenvironment [13, 49]. The pretreatments have become the potential attractive indicator of prognosis for estimating the inflammatory response and outcomes of cancer patients. However, these two cell types play different roles in the inflammation response and cancer progression [50]. Neutrophils promote the proliferation and survival and of cancer cells by collecting more inflammation mediators such as interleukin-1, interleukin-6, vascular endothelial growth factor and tumour necrosis factor, and large number of neutrophils suppress cytolytic activity of lymphocytes, natural killer cells and T-cell proliferation function [51, 52]. However, lymphocytes have a vital role in the immune defense against tumour cells, which inhibit tumor cell proliferation and metastases [53, 54]. As an indicator of concisely reflecting the balance between neutrophils and immunocytes, NLR is a promising prognostic factor [22, 23, 26]. Additionally, it is a convenient and cost-effective and reproducible index to measure the NLR in clinical practice, which makes NLR an attractive biomarker for OC prognostication.

Although one recent meta-analysis have reported that high NLR is associated with an adverse OS and EDS in patients with gynecologic malignancies [55], OC has not been discussed emphatically and additional original studies with larger sample sizes have been published since then [40, 42, 44, 47], which led to its limitations. To provide a more precise assessment of the prognostic role of NLR for patients with OC, we performed a comprehensive meta-analysis by including the most recent and relevant articles. In the present study, 16 studies with 4,910 patients were included to calculate pooled HR. The results demonstrated that elevated pretreatment NLR was significantly related to poor OS and PFS in patients with OC. These findings are similar to the previous meta-analyzes, which also evaluated the prognostic value of NLR in patients with other malignancies [11, 14–17, 19, 21–24, 27, 28, 30, 31, 56] or gynecologic cancers. Due to the extreme heterogeneity between studies, subgroup analyses by ethnicity, sample size, histologic types, primary treatment, cut-off value of NLR, analysis method and NOS score did not show obviously significant change in the main results. Moreover, our meta-regression analysis showed that none of the variables listed above contributed to the heterogeneity. Furthermore, in terms of the correlations between NLR and clinicopathological characteristics, we did not conduct quantitative synthesis because of a relatively small amount of studies and sample size. Because of these deficiencies, although we are able to conclude that the pooled estimate of available studies indicates that a higher NLR is associated with a poor OC prognosis, whether the NLR can serve prospectively as a clinical marker of prognosis will need further investigation. In addition, the publication bias was not significant, indicating that the results of our meta-analysis are reliable. To the best of our knowledge, this is the first PROSPERO meta-analysis exploring the prognostic effects of increased NLR in OS and PFS in patients with OC.

This meta-analysis has several limitations that should be acknowledged. First, a large part of included studies were retrospective analysis and thus more susceptible to the bias in data selection. Second, the patients in most of included publications were from Asian ethnicity, which may cause selection bias for general population. Third, some studies included all pathological types of OC patients, which may cause heterogeneity and interfere with the execution of subgroup analysis and meta-regression. Fourth, the cut-off values for determining elevated NLR in each individual study were inconsistent, which may cause the heterogeneity to some extent. Finally, some studies provided the corresponding HRs but their 95% CIs could only be retrieved from univariate analysis or calculated from Kaplan-Meier curves rather than from multivariate analysis, which may impair the accuracy of the pooled estimates.

In conclusion, our meta-analysis demonstrated that elevated NLR by pretreatment was closely associated with poor survival outcome in OC patients. However, attention should be paid cautiously due to the limitations listed above. To better understand the role of NLR in the prognosis of OC patients, more large-scale and well-designed investigations should be conducted.

## MATERIALS AND METHODS

### Literature search

The protocol of this review was registered with PROSPERO (No. CRD 42016052250) (Supplementary Table 1). This analysis was carried out in accordance with the Preferred Reporting Items for Systematic Reviews and Meta-Analyses (PRISMA) guidelines (Supplementary Table 2). We conducted thorough literature search using the PubMed, Embase and Cochrane Library for related articles that evaluated the prognostic value of NLR for ovarian cancer. The literature search was last updated on January 31, 2017. The following medical subject headings (MeSH) and keywords employed in the literature search included: (‘ovarian neoplasms’ OR ‘ovarian carcinoma’ OR ‘ovarian tumor’ OR ‘ovarian cancer’) AND (‘NLR’ OR ‘neutrophil to lymphocyte ratio’ OR ‘neutrophil-to-lymphocyte ratio’ OR ‘neutrophil lymphocyte ratio’) AND (‘prognosis’ OR ‘prognostic’ OR ‘survival’ OR ‘outcome’ OR ‘mortality’). There were no restrictions in language or publication date. In addition to the literatures searched from the above sources, the articles listed in the reference lists of the reviewed articles were also manually reviewed to obtain additional eligible studies.

### Inclusion and exclusion criteria

Selection criteria studies included in the meta-analysis need to meet the following criteria: (1) evaluated the prognostic value of NLR for OC; (2) patients with a pathologically confirmed diagnosis of OC; (2) the value of NLR was acquired from a peripheral blood before treatment; (3) HRs and 95% CIs for NLR in OS and (or) PFS were reported, or the data sufficient to estimate the HR and 95% CIs in studies; (4) the cut-off value of NLR was clearly reported. A study must meet all four inclusion criteria for inclusion. The exclusion criteria were: (1) letters, conference abstracts or review articles; (2) animal studies, non-clinical studies or case reports; (3) studies had overlapping or duplicate data; (4) no access to the full text for quality assessment and insufficient data to estimate HRs and 95% CIs; (4) did not present the cut-off value for elevated NLR.

### Data extraction and quality assessment

Eligible publications were reviewed by two investigators independently (Q Zhou and Z He) and the relevant data were extracted in a standardized table. The following data was extracted: surname of the first author’s surname, publication year, study country, ethnicity, duration, sample size, subtype of OC, tumor stage, tumor grade, treatment method, cut-off value defining elevated NLR and HRs with corresponding 95% CIs for PFS/RFS and(or) OS. The disagreements were resolved by consulting the third investigator (Li Hong).

The quality of each included study was assessed independently by two investigators assessed according to the Newcastle-Ottawa Scale (NOS) (http://www.ohri.ca/programs/clinical_epidemiology/oxford.asp) [57]. The NOS consists of three parameters of quality: selection (0-4 points), comparability (0-2 points), and outcome assessment (0-3 points). The maximum possible score is 9 points and NOS scores ≥7 are considered as high-quality studies. In the case of conflicting evaluations, a third investigator (Li Hong) was consulted, and disagreement was resolved by multilateral discussion.

### Statistical analysis

All data were analyzed using STATE 12.0 software (State Corporation, College Station, TX, USA), and all P values were two-sided. HRs and 95% CIs for OS and PFS were directly obtained from individual articles or calculated from indirect data according to the methods illustrated by Tierney et al [58]. When analyzing the relationship between NLR and clinicopathological factors, odds ratios (OR) and 95% CI were synthesized as the effective value. The heterogeneity among studies was estimated with the χ2-based Q test and Higgins’*I^2^* statistic. A *p*-value < 0.1 for the Q-test or *I^2^*>50% indicated significant heterogeneity, and the random-effects model was used, otherwise, the fixed-effects model was used. Sources of inter-study heterogeneity were explored using subgroup analysis. Sensitivity analyses were conducted to evaluate the stability of the results. Publication bias of literatures was evaluated using Begg’s funnel plot and Egger’s test.

## SUPPLEMENTARY MATERIALS TABLES







## References

[1] Siegel RL, Miller KD, Jemal A (2017). Cancer Statistics, 2017. CA Cancer J Clin.

[2] Torre LA, Bray F, Siegel RL, Ferlay J, Lortet-Tieulent J, Jemal A (2015). Global cancer statistics, 2012. CA Cancer J Clin.

[3] Smith RA, Andrews KS, Brooks D, Fedewa SA, Manassaram-Baptiste D, Saslow D, Brawley OW, Wender RC (2017). Cancer screening in the United States, 2017: A review of current American Cancer Society guidelines and current issues in cancer screening. CA Cancer J Clin.

[4] Kim SJ, Rosen B, Fan I, Ivanova A, McLaughlin JR, Risch H, Narod SA, Kotsopoulos J (2017). Epidemiologic factors that predict long-term survival following a diagnosis of epithelial ovarian cancer. Br J Cancer.

[5] Soletormos G, Duffy MJ, Othman Abu Hassan S, Verheijen RH, Tholander B, Bast RC, Gaarenstroom KN, Sturgeon CM, Bonfrer JM, Petersen PH, Troonen H, CarloTorre G, Kanty Kulpa J (2016). Clinical use of cancer biomarkers in epithelial ovarian cancer: updated guidelines from the european group on tumor markers. Int J Gynecol Cancer.

[6] Grivennikov SI, Greten FR, Karin M (2010). Immunity, inflammation, and cancer. Cell.

[7] Elinav E, Nowarski R, Thaiss CA, Hu B, Jin C, Flavell RA (2013). Inflammation-induced cancer: crosstalk between tumours, immune cells and microorganisms. Nat Rev Cancer.

[8] Fernandes JV, Cobucci RN, Jatoba CA, Fernandes TA, de Azevedo JW, de Araujo JM (2015). The role of the mediators of inflammation in cancer development. Pathol Oncol Res.

[9] Proctor MJ, Morrison DS, Talwar D, Balmer SM, Fletcher CD, O'Reilly DS, Foulis AK, Horgan PG, McMillan DC (2011). A comparison of inflammation-based prognostic scores in patients with cancer. A Glasgow Inflammation Outcome Study. Eur J Cancer.

[10] McMillan DC (2009). Systemic inflammation, nutritional status and survival in patients with cancer. Curr Opin Clin Nutr Metab Care.

[11] Wu Y, Fu X, Zhu X, He X, Zou C, Han Y, Xu M, Huang C, Lu X, Zhao Y (2011). Prognostic role of systemic inflammatory response in renal cell carcinoma: a systematic review and meta-analysis. J Cancer Res Clin Oncol.

[12] Shrotriya S, Walsh D, Bennani-Baiti N, Thomas S, Lorton C (2015). C-reactive protein is an important biomarker for prognosis tumor recurrence and treatment response in adult solid tumors: a systematic review. PLoS One.

[13] Buergy D, Wenz F, Groden C, Brockmann MA (2012). Tumor-platelet interaction in solid tumors. Int J Cancer.

[14] Templeton AJ, McNamara MG, Seruga B, Vera-Badillo FE, Aneja P, Ocana A, Leibowitz-Amit R, Sonpavde G, Knox JJ, Tran B, Tannock IF, Amir E (2014). Prognostic role of neutrophil-to-lymphocyte ratio in solid tumors: a systematic review and meta-analysis. J Natl Cancer Inst.

[15] Nishijima TF, Muss HB, Shachar SS, Tamura K, Takamatsu Y (2015). Prognostic value of lymphocyte-to-monocyte ratio in patients with solid tumors: a systematic review and meta-analysis. Cancer Treat Rev.

[16] Templeton AJ, Ace O, McNamara MG, Al-Mubarak M, Vera-Badillo FE, Hermanns T, Seruga B, Ocana A, Tannock IF, Amir E (2014). Prognostic role of platelet to lymphocyte ratio in solid tumors: a systematic review and meta-analysis. Cancer Epidemiol Biomarkers Prev.

[17] Paramanathan A, Saxena A, Morris DL (2014). A systematic review and meta-analysis on the impact of pre-operative neutrophil lymphocyte ratio on long term outcomes after curative intent resection of solid tumours. Surg Oncol.

[18] Huang QT, Man QQ, Hu J, Yang YL, Zhang YM, Wang W, Zhong M, Yu YH (2017). Prognostic significance of neutrophil-to-lymphocyte ratio in cervical cancer: a systematic review and meta-analysis of observational studies. Oncotarget.

[19] Wu J, Chen M, Liang C, Su W (2017). Prognostic value of the pretreatment neutrophil-to-lymphocyte ratio in cervical cancer: a meta-analysis and systematic review. Oncotarget.

[20] Ethier JL, Desautels D, Templeton A, Shah PS, Amir E (2017). Prognostic role of neutrophil-to-lymphocyte ratio in breast cancer: a systematic review and meta-analysis. Breast Cancer Res.

[21] Wei B, Yao M, Xing C, Wang W, Yao J, Hong Y, Liu Y, Fu P (2016). The neutrophil lymphocyte ratio is associated with breast cancer prognosis: an updated systematic review and meta-analysis. Onco Targets Ther.

[22] Liu JF, Ba L, Lv H, Lv D, Du JT, Jing XM, Yang NJ, Wang SX, Li C, Li XX (2016). Association between neutrophil-to-lymphocyte ratio and differentiated thyroid cancer: a meta-analysis. Sci Rep.

[23] Yang HB, Xing M, Ma LN, Feng LX, Yu Z (2016). Prognostic significance of neutrophil-lymphocyteratio/platelet-lymphocyteratioin lung cancers: a meta-analysis. Oncotarget.

[24] Tsai PL, Su WJ, Leung WH, Lai CT, Liu CK (2016). Neutrophil-lymphocyte ratio and CEA level as prognostic and predictive factors in colorectal cancer: a systematic review and meta-analysis. J Cancer Res Ther.

[25] Cao J, Zhu X, Zhao X, Li XF, Xu R (2016). Neutrophil-to-lymphocyte ratio predicts PSA response and prognosis in prostate cancer: a systematic review and meta-analysis. PLoS One.

[26] Qi X, Li J, Deng H, Li H, Su C, Guo X (2016). Neutrophil-to-lymphocyte ratio for the prognostic assessment of hepatocellular carcinoma: a systematic review and meta-analysis of observational studies. Oncotarget.

[27] Na N, Yao J, Cheng C, Huang Z, Hong L, Li H, Qiu J (2016). Meta-analysis of the efficacy of the pretreatment neutrophil-to-lymphocyte ratio as a predictor of prognosis in renal carcinoma patients receiving tyrosine kinase inhibitors. Oncotarget.

[28] Xin-Ji Z, Yong-Gang L, Xiao-Jun S, Xiao-Wu C, Dong Z, Da-Jian Z (2015). The prognostic role of neutrophils to lymphocytes ratio and platelet count in gastric cancer: A meta-analysis. Int J Surg.

[29] Cheng H, Long F, Jaiswar M, Yang L, Wang C, Zhou Z (2015). Prognostic role of the neutrophil-to-lymphocyte ratio in pancreatic cancer: a meta-analysis. Sci Rep.

[30] Yodying H, Matsuda A, Miyashita M, Matsumoto S, Sakurazawa N, Yamada M, Uchida E (2016). Prognostic significance of neutrophil-to-lymphocyte ratio and platelet-to-lymphocyte ratio in oncologic outcomes of esophageal cancer: a systematic review and meta-analysis. Ann Surg Oncol.

[31] Yang X, Huang Y, Feng JF, Liu JS (2015). Prognostic significance of neutrophil-to- lymphocyte ratio in esophageal cancer: a meta-analysis. Onco Targets Ther.

[32] Cengiz FP, Emiroglu N, Ozkaya DB, Bahali AG, Su O, Onsun N (2017). Prognostic evaluation of neutrophil/lymphocyte ratio in patients with mycosis fungoides. Ann Clin Lab Sci.

[33] Asher V, Lee J, Innamaa A, Bali A (2011). Preoperative platelet lymphocyte ratio as an independent prognostic marker in ovarian cancer. Clin Transl Oncol.

[34] Badora-Rybicka A, Nowara E, Starzyczny-Slota D (2016). Neutrophil-to-lymphocyte ratio and platelet-to-lymphocyte ratio before chemotherapy as potential prognostic factors in patients with newly diagnosed epithelial ovarian cancer. ESMO Open.

[35] Cho H, Hur HW, Kim SW, Kim SH, Kim JH, Kim YT, Lee K (2009). Pre-treatment neutrophil to lymphocyte ratio is elevated in epithelial ovarian cancer and predicts survival after treatment. Cancer Immunol Immunother.

[36] Eo WK, Chang HJ, Kwon SH, Koh SB, Kim YO, Ji YI, Kim HB, Lee JY, Suh DS, Kim KH, Chang IJ, Kim HY, Chang SC (2016). The lymphocyte-monocyte ratio predicts patient survival and aggressiveness of ovarian cancer. J Cancer.

[37] Feng Z, Wen H, Bi R, Ju X, Chen X, Yang W, Wu X (2016). Preoperative neutrophil-to-lymphocyte ratio as a predictive and prognostic factor for high-grade serous ovarian cancer. PLoS One.

[38] Gungorduk K, Ertas IE, Ozdemir A, Akkaya E, Telli E, Taskin S, Gokcu M, Guzel AB, Oge T, Akman L, Toptas T, Solmaz U, Dogan A (2015). Prognostic significance of retroperitoneal lymphadenectomy, preoperative neutrophil lymphocyte ratio and platelet lymphocyte ratio in primary fallopian tube carcinoma: a multicenter study. Cancer Res Treat.

[39] Kim HS, Choi HY, Lee M, Suh DH, Kim K, No JH, Chung HH, Kim YB, Song YS (2016). Systemic inflammatory response markers and CA-125 levels in ovarian clear cell carcinoma: a center cohort study. Cancer Res Treat.

[40] Li Z, Hong N, Robertson M, Wang C, Jiang G (2017). Preoperative red cell distribution width and neutrophil-to-lymphocyte ratio predict survival in patients with epithelial ovarian cancer. Sci Rep.

[41] Miao Y, Yan Q, Li S, Li B, Feng Y (2016). Neutrophil to lymphocyte ratio and platelet to lymphocyte ratio are predictive of chemotherapeutic response and prognosis in epithelial ovarian cancer patients treated with platinum-based chemotherapy. Cancer Biomark.

[42] Paik ES, Shim M, Choi HJ, Lee YY, Kim TJ, Choi CH, Lee JW, Kim BG, Bae DS (2016). Preoperative multiplication of neutrophil and monocyte counts as a prognostic factor in epithelial ovarian cancer. Cancer Biomark.

[43] Thavaramara T, Phaloprakarn C, Tangjitgamol S, Manusirivithaya S (2011). Role of neutrophil to lymphocyte ratio as a prognostic indicator for epithelial ovarian cancer. J Med Assoc Thai.

[44] Wang Y, Liu P, Xu Y, Zhang W, Tong L, Guo Z, Ni H (2015). Preoperative neutrophil-to-lymphocyte ratio predicts response to first-line platinum-based chemotherapy and prognosis in serous ovarian cancer. Cancer Chemother Pharmacol.

[45] Wang YQ, Jin C, Zheng HM, Zhou K, Shi BB, Zhang Q, Zheng FY, Lin F (2016). A novel prognostic inflammation score predicts outcomes in patients with ovarian cancer. Clin Chim Acta.

[46] Williams KA, Labidi-Galy SI, Terry KL, Vitonis AF, Welch WR, Goodman A, Cramer DW (2014). Prognostic significance and predictors of the neutrophil-to-lymphocyte ratio in ovarian cancer. Gynecol Oncol.

[47] Zhang WQ, Hao Q (2014). Preoperative blood neutrophil to lymphocyte ratio as an independent prognostic predictor for epithelial ovarian cancer. Chin J Clin Oncol.

[48] Zhang WW, Liu KJ, Hu GL, Liang WJ (2015). Preoperative platelet/lymphocyte ratio is a superior prognostic factor compared to other systemic inflammatory response markers in ovarian cancer patients. Tumour Biol.

[49] Menon S, Shin S, Dy G (2016). Advances in cancer immunotherapy in solid tumors. Cancers (Basel).

[50] Faria SS, Fernandes PC, Silva MJ, Lima VC, Fontes W, Freitas-Junior R, Eterovic AK, Forget P (2016). The neutrophil-to-lymphocyte ratio: a narrative review. Ecancermedicalscience.

[51] Nicolas-Avila JA, Adrover JM, Hidalgo A (2017). Neutrophils in homeostasis, immunity, and cancer. Immunity.

[52] Uribe-Querol E, Rosales C (2015). Neutrophils in cancer: two sides of the same coin. J Immunol Res.

[53] Criscitiello C, Esposito A, Trapani D, Curigliano G (2016). Prognostic and predictive value of tumor infiltrating lymphocytes in early breast cancer. Cancer Treat Rev.

[54] Mao Y, Qu Q, Chen X, Huang O, Wu J, Shen K (2016). The prognostic value of tumor-infiltrating lymphocytes in breast cancer: a systematic review and meta-analysis. PLoS One.

[55] Ethier JL, Desautels DN, Templeton AJ, Oza A, Amir E, Lheureux S (2017). Is the neutrophil-to-lymphocyte ratio prognostic of survival outcomes in gynecologic cancers? A systematic review and meta-analysis. Gynecol Oncol.

[56] Teng JJ, Zhang J, Zhang TY, Zhang S, Li BS (2016). Prognostic value of peripheral blood lymphocyte-to-monocyte ratio in patients with solid tumors: a meta-analysis. Onco Targets Ther.

[57] Stang A (2010). Critical evaluation of the Newcastle-Ottawa scale for the assessment of the quality of nonrandomized studies in meta-analyses. Eur J Epidemiol.

[58] Tierney JF, Stewart LA, Ghersi D, Burdett S, Sydes MR (2007). Practical methods for incorporating summary time-to-event data into meta-analysis. Trials.

