# Supplementary material for: Prognostic significance of neutrophil to lymphocyte ratio in ovarian cancer: evidence from 4,910 patients
Source: Oncotarget. 2017 Aug 10;8(40):68938–49. doi: 10.18632/oncotarget.20196 (PMC5620309; doi:10.18632/oncotarget.20196)
Supplement: Supplementary file 1 [file oncotarget-08-68938-s001.pdf]

Prognostic significance of neutrophil to lymphocyte ratio in ovarian cancer: evidence from 4,910 patients

SUPPLEMENTARY MATERIALS

Supplementary Table 1: The protocol of this review was registered with PROSPERO (No. CRD 42016052250).

See Supplementary File 1

Supplementary Table 2: PRISMA Checklist. (DOC).

See Supplementary File 2
